# Remodelling of deformities and resolution of shortening in congenital posteromedial bowing of the tibia

**DOI:** 10.1186/s13018-025-06409-4

**Published:** 2025-11-14

**Authors:** Siddarth Kamath, Kumar Amerendra Singh, Siddardha Moka, Hitesh Shah

**Affiliations:** Department of Paediatric Orthopaedics, Kasturba Medical College, Manipal, Manipal Academy of Higher Education (MAHE), Manipal, Karnataka India

**Keywords:** Congenital posteromedial bowing of the tibia, Deformity correction, Limb lengthening, Limb length equalization, Estimation of limb shortening at maturity

## Abstract

**Background:**

In congenital posteromedial bowing of the tibia, the remodeling of triplanar (medial, posterior and oblique) deformities, the resolution of tibial shortening and their correlation with each other have not been studied extensively. Additionally, the age at which tibial shortening at maturity can be accurately predicted under these conditions is not known. This study aimed to evaluate the natural history of tri-planar deformity remodeling, the resolution of tibial shortening and the difference between tibial shortening predicted by the multiplier method at first presentation and subsequent follow-up.

**Methods:**

All consecutive children with congenital posteromedial bowing of the tibia (with a minimum of 2 years of follow-up) were prospectively evaluated. The severity of the bow was measured with diaphyseal and interphyseal (antero-posterior, lateral) angles. The percentage shortening (length of affected/normal side × 100) of the tibia was estimated at presentation and every year. The oblique plane deformity was calculated as per the trigonometric model. The multiplier method was used at presentation and at every follow-up to estimate tibial shortening at maturity, and the significant differences between the values were analyzed. Differences in the rates of deformity remodeling and the resolution of tibial shortening were compared by the Wilcoxon test. Spearman correlation was performed to assess the correlation between the degree of deformity remodeling and the resolution of tibial shortening.

**Results:**

Fifty-one children (21 boys, 30 girls) with a median duration of follow-up of 48 months were studied. The rate of remodeling of the posterior diaphyseal bow was greater than that of the median diaphyseal and oblique diaphyseal bows. The median rate of remodeling of the diaphyseal bows and interphyseal angles in the first year of life halved its value in subsequent years, and minimal remodeling was noted after 4–6 years of age. A total of 20% of tibial shortening at presentation resolved to 13% shortening at 6 years of age. There was no significant difference in tibial shortening estimated at maturity using the multiplier method after two years of age.

**Conclusion:**

Remodeling of the deformity and resolution of shortening provide better insight into deformity correction and planning for limb length equalization.

## Introduction

Congenital posteromedial bowing of the tibia (CPMBT) is a rare, benign, and partially self-resolving deformity of the tibia [1].

In the CPMBT, the posterior and medial tibial bows are known to remodel on the basis of Wolffes’ law and Heuter–Volkans’ law [3]. However, the remodelling process is not always complete, with some residual deformities persisting in some children. There are various opinions about the remodelling of deformities: the posterior bow remodels faster than the medial bow does [2–5], or both bows resolve at a parallel rate [6]. The remodelling of deformities occurs rapidly up to 3 years of age as the number of osteoprogenitor cells decreases thereafter [3, 7, 8]. However, the exact amount to which the deformities resolve and the natural history of the remodelling of these deformities are not known.

Compared with that of the normal tibia, the proportionate percentage of tibial shortening in the limb involved with CMPBT is known to remain stable according to the type 1 Shapiro curve [7, 9]. However, the magnitude of tibial shortening increases as the child grows, ultimately requiring tibial length equalization procedures [3, 7, 9]. However, the age and extent to which tibial shortening resolves are not well established.

Some authors have established a correlation between the remodelling of deformities and the resolution of tibial shortening and reported that the degree of angulation is positively correlated with tibial shortening [5, 7]. However, others have reported that the resolution of shortening is correlated with the severity of the medial bow [3] or the posterior bow [4]. However, these parameters have not been studied extensively.

The management of tibial shortening depends on the predicted shortening at skeletal maturity. Pappa et al. measured the proportionate tibial length to estimate tibial shortening at maturity on the basis of the type 1 Shapiro curve [7]. They reported that the measurements were accurate as early as three years of age. The Green‒Anderson method and the Moseley method have been used to estimate tibial shortening at maturity [9, 10]. More recently, the multiplier method has become one of the most commonly used methods to determine the same [6, 9–11]. However, the appropriate age at which this method can be applied to predict tibial shortening accurately at skeletal maturity is not known.

Congenital posteromedial bowing of the tibia is often considered a biplanar deformity. In these biplanar deformities, the true plane of the deformity lies in the third plane, i.e., the oblique plane [6, 11]. The deformity in the oblique plane, its evolution, and its contribution to the remodelling of limb shortening have not been studied.

In this study, a large cohort of children with CPMBT with a minimum of two years of follow-up was studied to answer these questions. We hypothesized that the shortening resolved in CPMBT and that the resolution of shortening was related to the remodelling of deformities.

This study aimed to study the following:

1. The remodelling of the triplanar deformities and resolution of tibial shortening.
2. The correlation between the remodelling of deformities and tibial shortening.
3. The difference between tibial shortening predicted by the multiplier method at first presentation and subsequent follow-ups.

## Materials and methods

In a prospective study, all consecutive cases of congenital postero-medial bowing of the tibia that presented to our center over a thirty-year time period were studied.

We included all children with unilateral or bilateral involvement with a minimum of two years of follow-up from the first visit. Children with other causes of tibial bowing, incomplete radiographs or incomplete follow-up data were excluded.

### Assessment of deformity

All measurements were performed on computerized radiographic images by two independent investigators using the Digimizer image analysis software (version 4.1.1.0; MedCalc Software, Mariakerke, Belgium), as outlined below. All radiographs were taken in uniform ways to avoid magnification errors in the measurements.

**Fig. 1:**
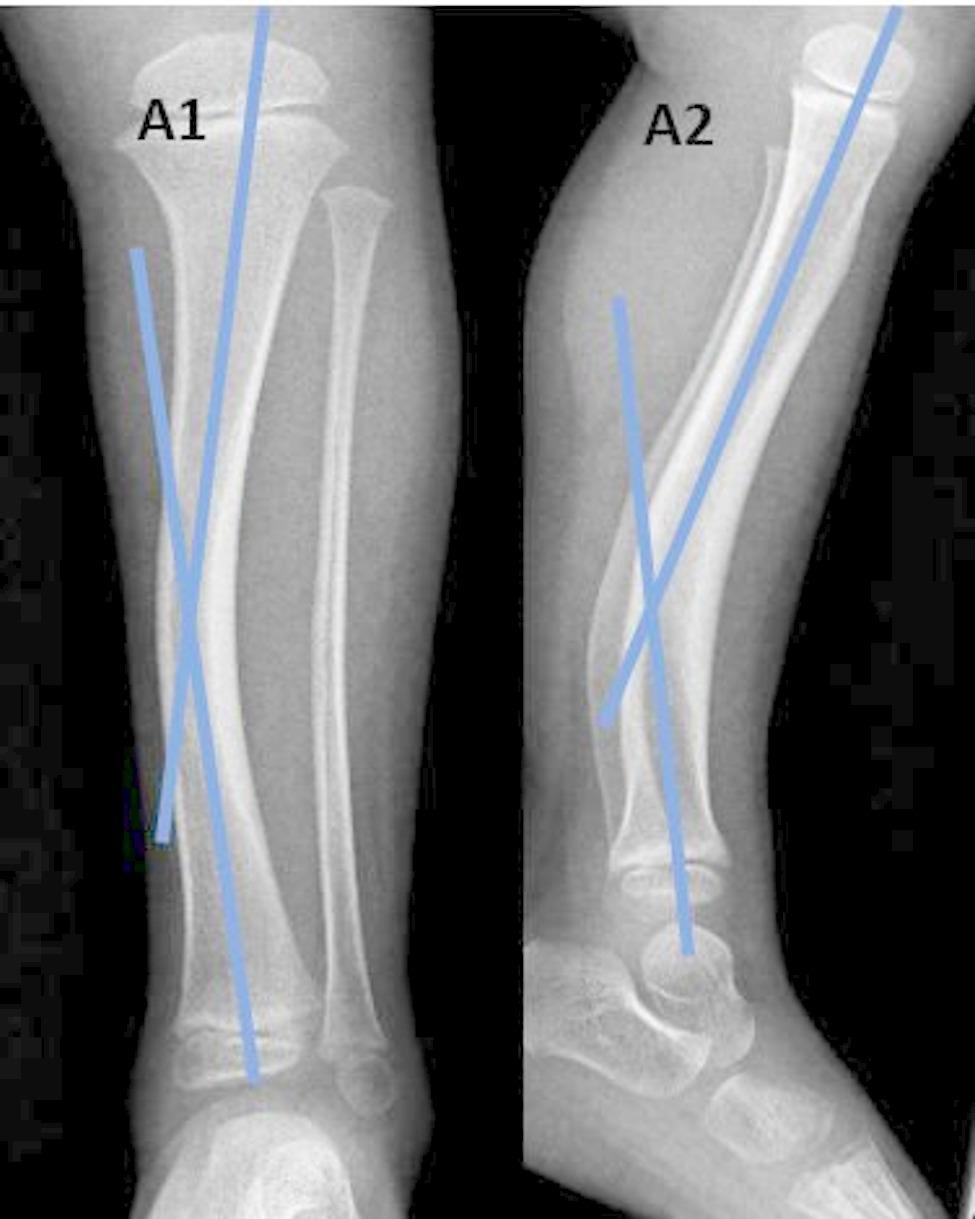
Measurement of diaphyseal bows, A1: Medial diaphyseal bow, A2: Posterior diaphyseal bow

**Fig. 2:**
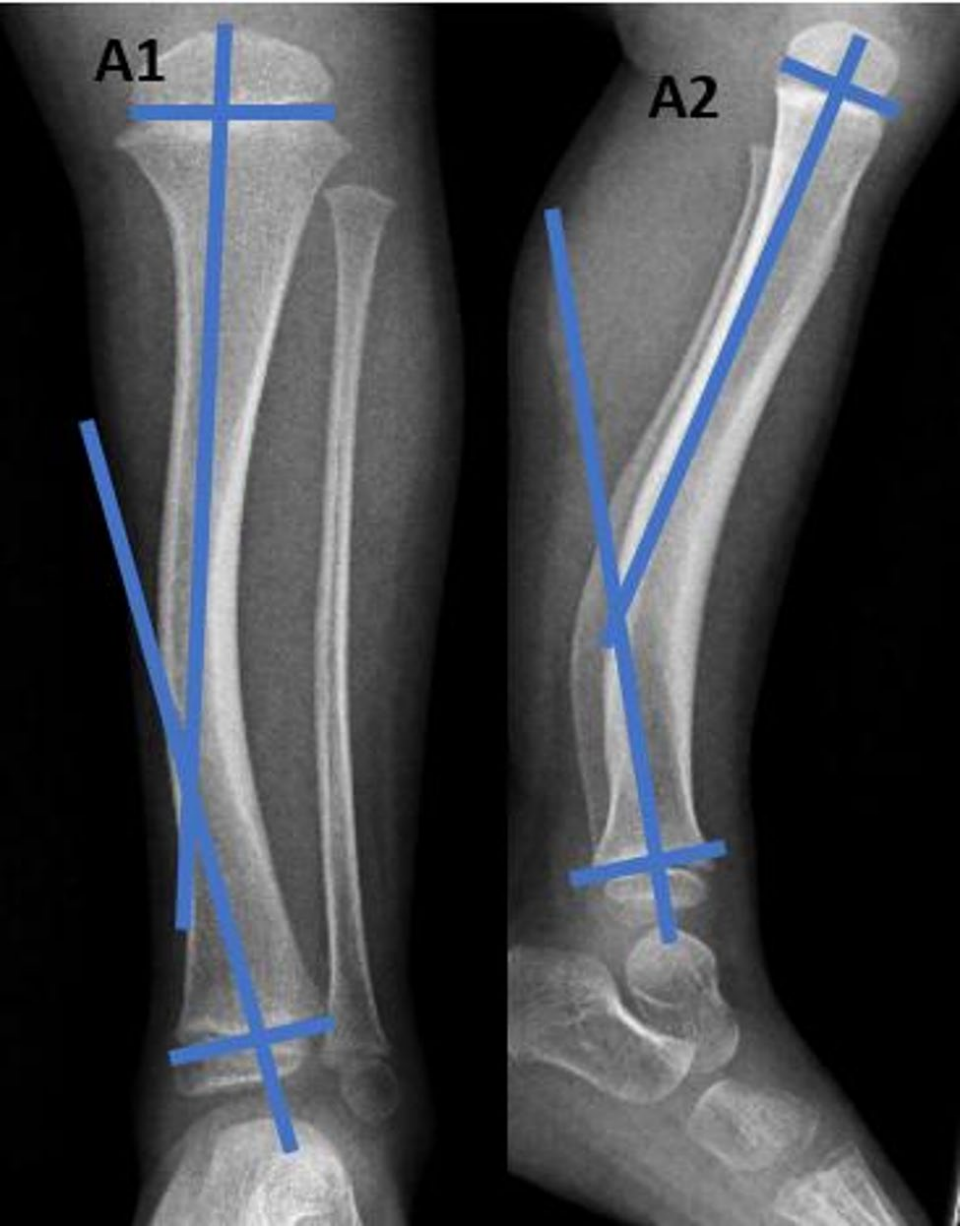
Measurement of interphyseal angles: A1: Interphyseal angle in the antero-posterior view; A2: Interphyseal angle in the lateral view

1. The deformity was measured separately on true anteroposterior and lateral radiographs in two ways:

- *The diaphyseal bow* (medial and posterior) (Fig. 1).
- *The inter-physeal angle* (inter-physeal anteroposterior, inter-physeal lateral) (Fig. 2).

These measurements were performed at presentation and at every follow-up until any surgical intervention altering the natural course of re-modelling of the deformity was performed.

2. On the basis of the values of the medial and posterior bows, the bow in the *oblique plane* was calculated using a *mathematical formula* [12] :

$$Oblique\;plane\;\left( {degrees} \right) = ArcTan\,\sqrt {\left( {Tan\;Medial} \right)^{2} + \left( {Tan\;Posterior} \right)^{2} }$$

3. The rate of remodelling of the diaphyseal and physeal deformities at presentation and subsequent follow-ups was calculated as follows (in degrees/month):

$$\frac{{{\text{First}}\;{\text{value}}\;\left( {{\text{degrees}}} \right) - \,{\text{Second}}\;{\text{value}}\;\left( {{\text{degrees}}} \right)}}{{{\text{Time}}\;{\text{duration}}\;{\text{between}}\;{\text{the}}\;{\text{two}}\;{\text{values}}\;({\text{months)}}}}$$

4. In our center, normal tibial radiographs were obtained along with the affected tibial radiographs at presentation and all subsequent follow-ups to compare the tibial lengths. The magnitude of shortening of the affected tibia was calculated, and the difference in shortening between the affected and normal tibias was calculated as follows [3, 7]:

$$\frac{{{\text{Differences}}\;{\text{in}}\;{\text{the}}\;{\text{lengths}}\;{\text{of}}\;{\text{the}}\;{\text{tibias}}}}{{{\text{Length}}\;{\text{of}}\;{\text{the}}\;{\text{unaffected}}\;{\text{tibia}}}} \times 100$$

5. The magnitude of tibial shortening was predicted using the multiplier method at presentation and at every follow-up [11].

### Statistical analysis

All the statistical analyses were performed using SPSS for Windows, and a *p* value < 0.05 was considered significant. The interclass and intraclass reliability were assessed using the interclass correlation coefficient. The Wilcoxon signed-rank test was used to test the statistical significance between the median bow at presentation and at the final follow-up. The Friedman test was used to assess the statistical significance between the rates of remodelling. The Spearman correlation coefficient was used to assess the correlation between the rate of remodelling of deformities and the rate of correction of limb shortening.

## Results

### Demography

Between 2000 and 2024, 118 children presented with congenital posteromedial bowing of the tibia.

Fifty-one children (52 limbs) with a minimum follow-up of 2 years were included in the study. Among the 67 excluded children, 36 underwent surgery at an early age, which may have interfered with the outcomes of the study, and 31 had incomplete radiographic images (Table 1).

**Table 1:** Demographic details

Gender involvement (N = 51)	Male = 21	Female = 30	
Side involvement (N = 50)	Right = 21	Left = 29	Bilateral = 1
Median age at presentation	6 months (IQR 4–6.75 months, Range: 1–120 months)		
Median duration of follow-up	48 months (IQR 24–96 months, Range: 24–156 months)		
Median age at final follow-up	48 months (IQR 36–96 months, 30 -162 months)		

### Reliability of measurement (Tables 2 and 3)

**Table 2:** Interobserver reliability

Variable	ICC (lower bound − upper bound)	*p*
Medial bow	0.95 (0.93–0.97)	0.00
Posterior bow	0.96 (0.95–0.98)	0.00
IP angle AP view	0.97 (0.95–0.98)	0.00
IP angle Lateral view	0.94 (0.91–0.96)	0.00
Assessment of limb length discrepancy	0.85 (0.75–0.89)	0.00

**Table 3:** Intraobserver reliability

Variable	ICC (lower bound–upper bound)	*p*
Medial bow	0.98 (0.97–0.98)	0.00
Posterior bow	0.99 (0.98–0.99)	0.00
IP angle AP view	0.98 (0.96–0.98)	0.00
IP angle Lateral view	0.94 (0.90–0.96)	0.00
Assessment of limb length discrepancy	0.81 (0.71–0.82)	0.00

All radiological parameters were measured by two independent observers and by the same observer 2 months apart. All the parameters measured had good reproducibility.

### Overall remodelling of deformities (Fig. 3) and overall resolution of shortening (Figs. 4, 5, 6)

**Fig. 3:**
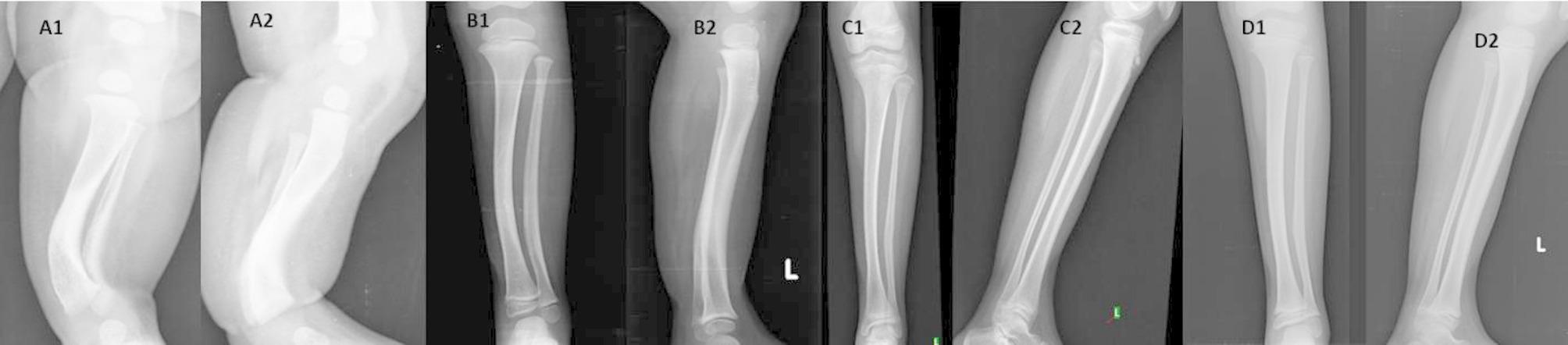
Showing complete remodeling of deformities. A1, A2- At presentation, showing a medial bow of 50 degrees and a posterior bow of 40 degrees. B1, B2- At 3 years, a medial bow of 30 degrees and a posterior bow of 20 degrees were observed. C1, C2- At 6 years, showing a medial bow of 10 degrees and a posterior bow of 4 degrees. D1, D2- At 12 years, a medial bow of 8 degrees and a posterior bow of 4 degrees were observed

**Fig. 4:**
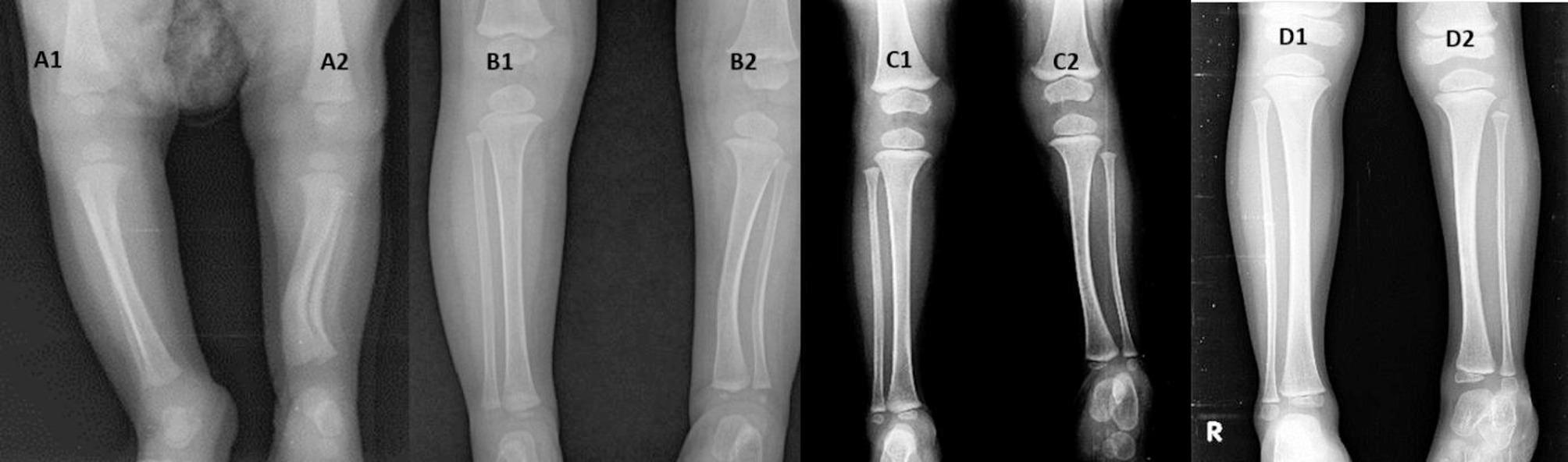
Showing remodeling of shortening. A1, A2- At 6 months, the measured shortening was 2.7 cm, and the proportionate shortening was 25%. B1, B2- At 2 years, the measured shortening was 3 cm, and the proportionate shortening was 18%. C1, C2- At 6 years, the measured shortening was 4 cm, and the proportionate shortening was 15%. D1, D2- At 8 years, the measured shortening was 4 cm, and the proportionate shortening was 13%

**Fig. 5:**
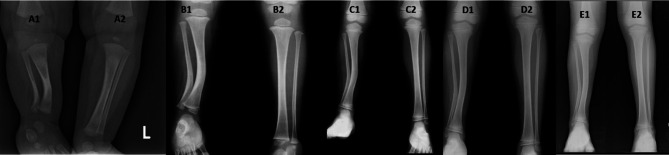
Showing the prediction of shortening via the multiplier method. A1,A2- At 6 months, a shortening of 2.5 cm was measured, with shortening rates of 25% and 8.3 cm estimated at maturity via the multiplier method. B1, B2- At 2 years, a shortening of 3 cm was measured, with shortening rates of 17% and 6 cm estimated at maturity via the multiplier method. C1, C2- At 4 years, a shortening of 3.5 cm was measured, with shortening rates of 15% and 6 cm estimated at maturity via the multiplier method. D1, D2- At 6 years, a shortening of 4 cm was measured, with shortening rates of 15% and 6 cm estimated at maturity via the multiplier method. E1,E2- At 8 years, a measured shortening of 5 cm, proportionate shortening of 15%, and 6 cm shortening estimated at maturity via the multiplier method

**Fig. 6:**
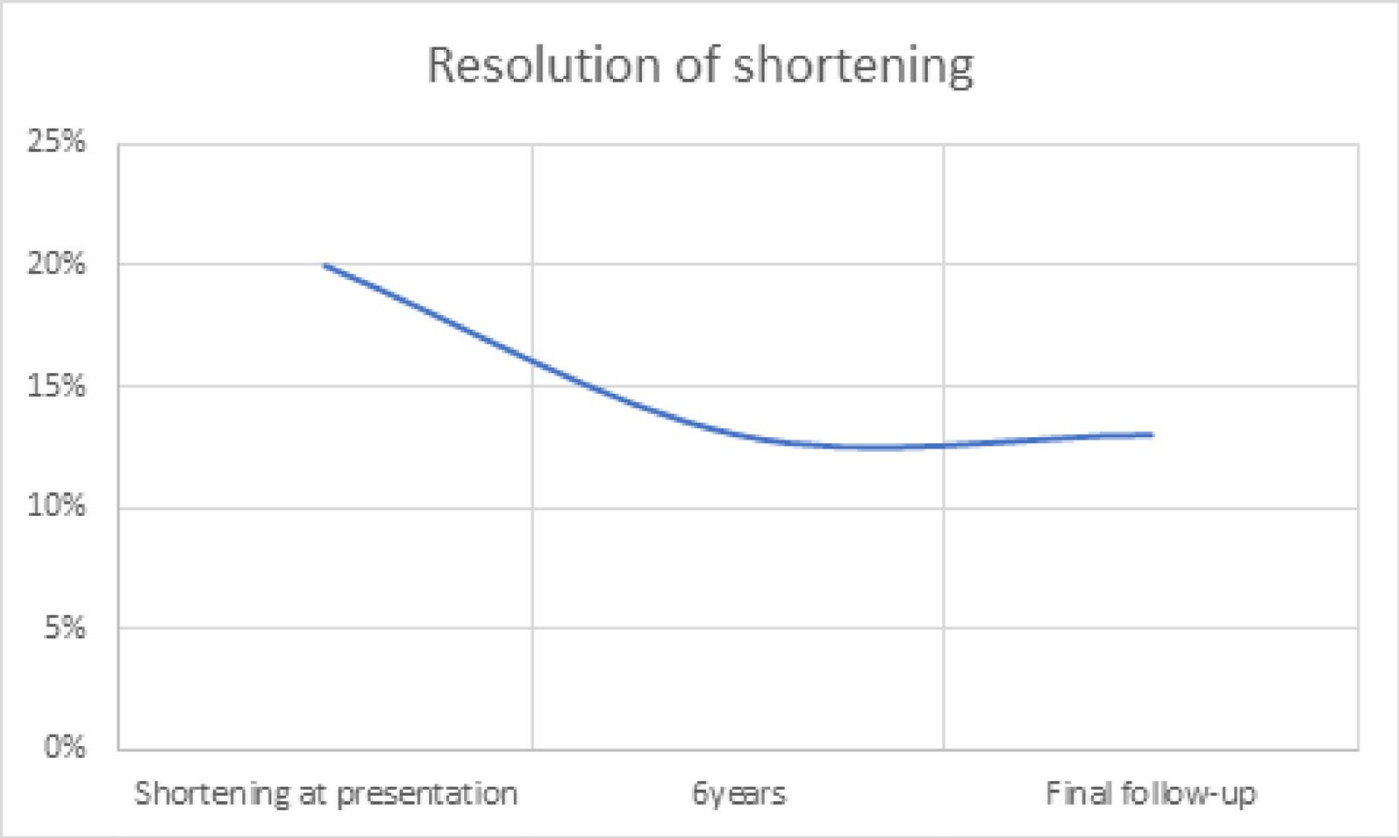
The percentage of shortening is plotted against age. The percentage shortening decreased from 20% at presentation to 13% at 6 years of age and remained constant thereafter

#### Antero-posterior plane (Table 4)

**Table 4:** Deformity resolution in the antero-posterior plane (values marked with * are statistically significant)

Bowing	Bowing at presentation Median (IQR) (degrees)	Bowing at the final follow-up Median (IQR) (degrees)	Rate of overall remodelling Median (IQR) (degrees/month)	Percentage correction Median (IQR) (Minmax)
Medial diaphyseal	40* (30.5–48)	17* (10–25.75)	0.41 (0.24–0.58)	60.3*(32.3–72.9) (Min 22.2–Max 86)
Interphyseal	35* (21.25–45)	12* (6–20)	(0.23–0.55)	65* (44.4–83.3) (Min 27.7–95)

At the final follow-up, the diaphyseal and interphyseal deformities had remodelled to 60% and 65% of their values at the first presentation, respectively. This observation was statistically significant.

###  Lateral plane (Table 5)

**Table 5:** Deformity resolution in the lateral plane (values marked with * are statistically significant)

Bowing	Bowing at presentation Median (IQR) (degrees)	Bowing at the final follow-up Median (IQR) (degrees)	Rate of overall remodelling Median (IQR) (degrees/month)	Percentage correction Median (IQR) (Min–max)
Posterior diaphyseal	35.5*(30–50.5)	10*(6.25–21.5)	0.60 (0.33–0.80)	66.6*(46.1–81.2) (Min 5.5−Max 94.1)
Interphyseal	36.5*(25–45.75)	6.5*(4–18)	(0.18–0.57)	73* (36.8–88.5) (Min 10- Max 94.7)

At the final follow-up, the median posterior diaphyseal bow had remodelled to 66%, and the median inter-physeal angle in the posterior plane had remodelled to 73% of its value at the first presentation. These observations were statistically significant.

###  Oblique plane (Table 6)

**Table 6:** Deformity resolution in the oblique plane (values marked with * are statistically significant)

Variable	Bowing at presentation Median (IQR) (degrees)	Bowing at the final follow-up Median (IQR) (degrees)	Rate of overall remodelling Median (IQR) (degrees/month)	Percentage correction Median (IQR) (Min–max)
Oblique diaphyseal	49*(40–58.75)	20*(13.25–31.75)	0.45 (0.32–0.69)	60.3* (30.5–71.4) (Min 11.1-Max 85.5)
Inter-physeal	44*(34–54)	16*(8–26.75)	0.45 (0.33–0.65)	61.9* (42.2–83.3) (Min 16.4-Max 94)

At the final follow-up, the diaphyseal and inter-physeal deformities in the oblique plane had remodeled to 60% of the initial value. This observation was statistically significant. The rates of remodeling of the diaphyseal and interphyseal deformities were similar.

###  Limb shortening (Table 7)

**Table 7:** Resolution of tibial shortening (values marked with * are statistically significant)

Shortening	At presentation Median (IQR)	At final follow-up Median (IQR)	Rate of overall resolution Median (IQR) (per month)	Percentage correction Median (IQR) (Min–max)
Magnitude of limb shortening (cm)	2*(2–3)	4*(3–5)	NA	40%* increase (25–54) (Min 16.6–Max 70)
% shortening	20*(15–25)	13*(11.25–17.75)	0.07 (0.02–0.15)	34%* (16.6–41.3) (Min 3.8–Max 6)

The median tibial shortening increased from 2 cm at presentation to 4 cm at the final follow-up. This corresponded to a 40% increase in shortening at the final follow-up. Although the magnitude increased, the percentage of shortening compared with that of the opposite side decreased from 20 to 13%. The median rate of remodeling was 0.07% per month. This corresponded to a 34% reduction in the percentage of tibial shortening at the final follow-up. These observations were statistically significant.

## Yearly rate of remodelling of deformities and yearly resolution of shortening

### Modelling of the diaphyseal bow and inter-physeal angle (Table 8, Fig. 7)

**Table 8:** Yearly rate of remodelling of deformities (degrees/month)

Plane	Antero-posterior Median (IQR) (degrees/month)		Lateral Median (IQR) (degrees/month)		Oblique Median (IQR) (degrees/month)		*p*
	Medial diaphyseal	Inter-physeal	Posterior diaphyseal	Inter-physeal	Diaphyseal	Inter-physeal	
< 1 year	1.33 (0.87–1.6)	1.16 (0.6–1.7)	1.66 (1.09–2)	1.11 (0.5–2)	1.50 (1.2–1.9)	1.36 (0.7–2.1)	< 0.01
1–2 years	0.66 (0.37–0.79)	0.58 (0.25–0.87)	0.66 (0.33–0.83)	0.66 (0.37–0.87)	0.66 (0.45–0.87)	0.83 (0.37–0.95)	< 0.01
2–3 years	0.29 (0.16–0.41)	0.41 (0.25–0.50)	0.41 (0.20–0.62)	0.41 (0.27–0.83)	0.45 (0.35–0.56)	0.54 (0.41–0.83)	< 0.01
3–4 years	0.29 (0.16–0.33)	0.37 (0.25–0.5)	0.25 (0.16–0.43)	0.37 (0.25–0.6)	0.29 (0.25–0.35)	0.58 (0.45–0.75)	NS
4–6 years	0.16 (0.08–0.29)	0.16 (0.04–0.25)	0.12 (0.08–0.20)	0.16 (0.04–0.25)	0.29 (0.12–0.33)	0.12 (0.12–0.33)	NS
> 6 years	0.12 (0.08–0.19)	0.08 (0.05–0.12)	0.08 (0.04–0.15)	0.06 (0.04–0.15)	0.16 (0.12–0.30)	0.12 (0.08–0.16)	NS

**Fig. 7:**
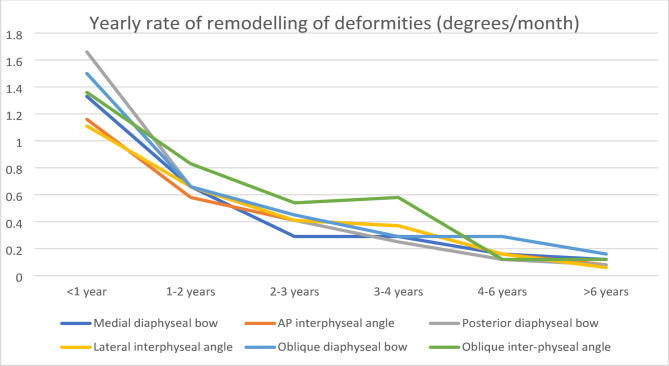
Yearly rate of remodeling of deformities (degrees/month)

The maximum rate of remodelling was observed in the first twelve months of life. The rate of remodelling of the posterior diaphyseal bow was the highest during this period. This rate was reduced to half of its value between 1 and 2 years. During the 2–3-year period, the rate of remodelling further decreased to half of its value. These observations were statistically significant.

After 3 years of age, the rate of remodelling decreased significantly up to 6 years of age. After 6 years of age, minimal remodelling of all three deformities was noted.

###  Resolution of tibial shortening (Table 9 and Fig. 8)

**Table 9:** Yearly rate of resolution of shortening

Age (years)	Median (IQR)(%/year)
< 1	0.40 (0.16–0.66)
1–2	0.16 (0.04–0.25)
2–3	0.08 (0.0–0.16)
3–4	0.04 (0.0–0.08)
4–6	0.02 (0.00–0.14)
> 6	0.008 (0.0–04)

**Fig. 8:**
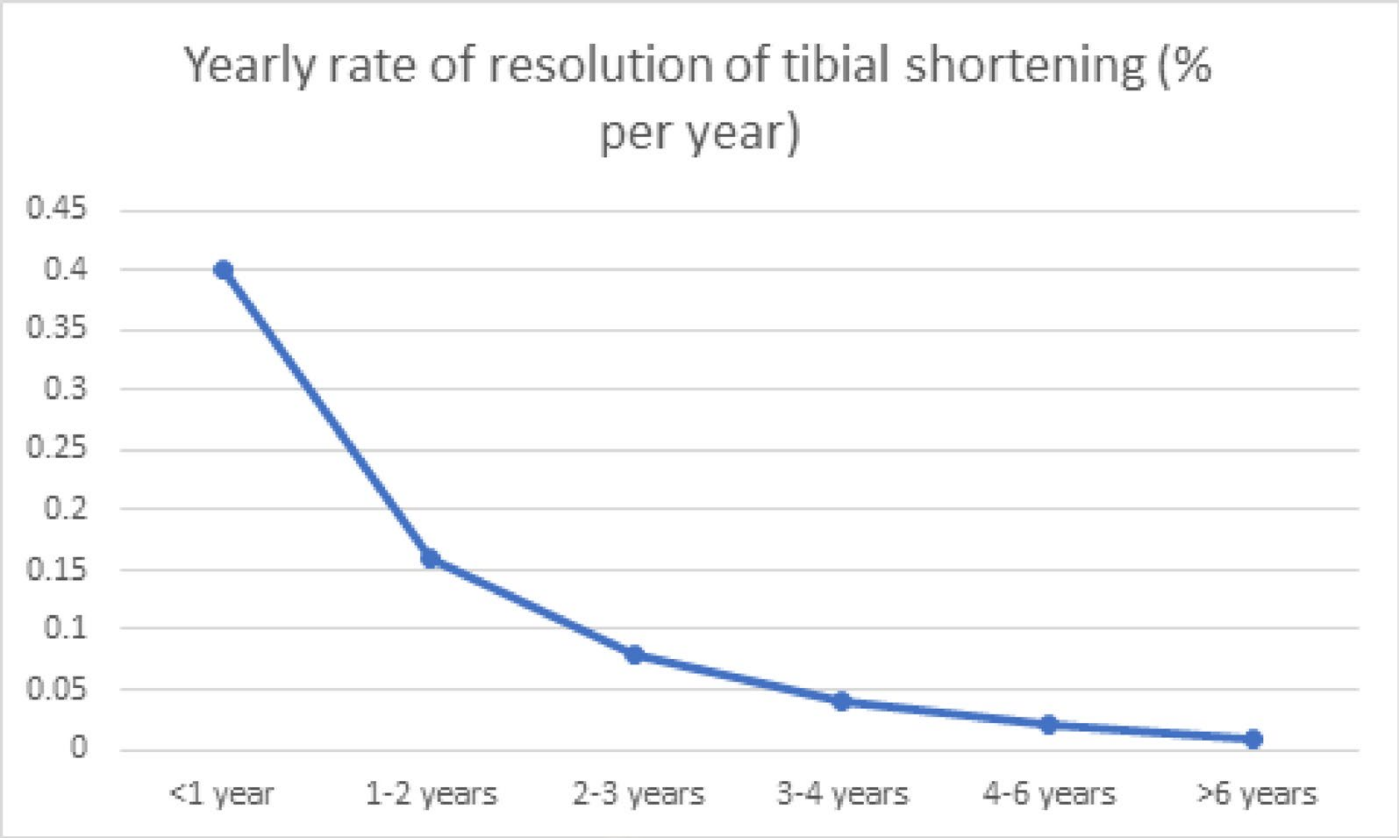
Yearly resolution of tibial shortening

The maximum rate of remodelling of percentage shortening was observed in the first year of life. This value was reduced to 50% of its value in the second year and further 50% in the third year. There was minimal remodelling of shortening after 6 years of age.

## Correlation between the rate of remodelling of deformities and the rate of correction of limb shortening (Table 10)

**Table 10:** Relationships between the rate of deformity remodelling and the degree of tibial shortening remodelling

Plane	Deformity	Spearman correlation-r
Antero-posterior	Medial diaphyseal	0.2
	Inter-physeal	0.2
Lateral	Posterior diaphyseal	0.2
	Inter-physeal	0.2
Oblique	Oblique diaphyseal	0.4
	Inter-physeal	0.4

There was a moderate correlation between the remodelling of the diaphyseal and inter-physeal deformities in the oblique plane and the resolution of the affected tibial shortening. There was a weak correlation between the rate of remodelling of the deformities in the other diaphyseal and inter-physeal planes and the correction of tibial shortening.

## Analysis of measured limb length shortening and its correlation with the multiplier method for predicting limb shortening at skeletal maturity (Tables 11, 12 and Fig. 9)

**Table 11:** Overall comparison between the measured and predicted shortening

Shortening	At presentation (Mean ± SD)	Final follow-up (Mean ± SD)	*p*
Actual shortening (cm)	2 ± 1.09	4 ± 1.4	0.00
Predicted shortening at maturity (cm)	8.7 ± 2.7	6.7 ± 2.3	0.00

**Table 12:** Yearly comparison between the measured and predicted shortening

Age (months)	Actual tibial shortening Mean ± SD (cm)	Predicted tibia shortening at maturity Mean ± SD (cm)	*p*
6 (n = 29)	2.2 ± 0.5	9.5 ± 2.1	0.00
12 (n = 31)	2.4 ± 0.75	7.7 ± 2.5	0.00
24 (n = 36)	2.9 + 0.8	7.5 ± 2.3	0.00
36 (n = 22)	3.2 ± 1.0	7.2 ± 2.3	0.63
48 (n = 12)	3.7 + 0.8	7 ± 2.2	0.84
72 (n = 8)	4.1 ± 0.35	6.7 ± 0.62	0.73
84 (n = 8)	4.1 ± 0.73	6.5 ± 1.27	0.71
96 (n = 8)	4.7 ± 1.07	6.5 ± 1.01	0.92
108 (n = 3)	4.7 ± 0.57	6.3 ± 0.75	NS
120 (n = 3)	5.1 ± 0.76	6.3 ± 1.15	NS
132 (n = 3)	5.3 ± 0.76	6.3 ± 0.91	NS

**Fig. 9:**
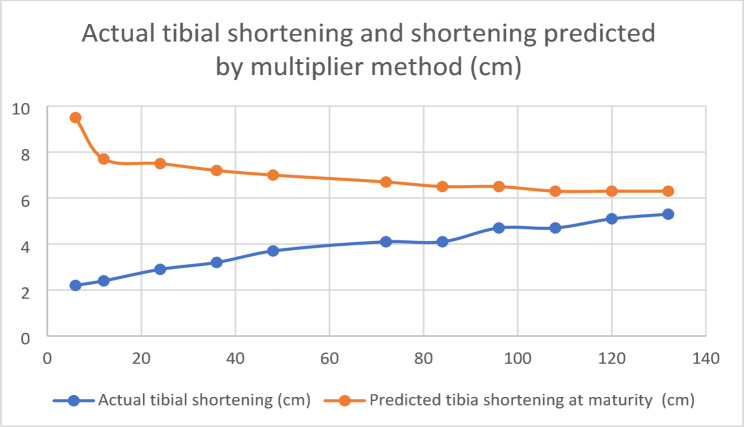
Actual shortening and shortening predicted by multipliers (in centimeters) plotted against age (in months)

There was a statistically significant difference in the mean measured shortening at presentation and at the final follow-up. The multiplier method was used to predict shortening at skeletal maturity at the first and all subsequent follow-ups. There was a statistically significant difference in the mean predicted tibial shortening between the first and final follow-ups.

The degree of predicted shortening was assessed at each follow-up and assessed separately. There was a statistically significant difference between the predicted limb shortening up to 24 months. However, after 24 months, no statistically significant difference was observed in the predicted limb shortening measured by the multiplier method.

## Discussion

### Demography and incidence

The exact incidence of congenital posteromedial tibia has not been accurately established. In this study, 118 children with CPMBT were studied over 24 years. This number corresponds to 4–5 cases every year at our institute. In the literature reviewed, this is the largest cohort of children with CPMBT.

In this cohort, one child had effects on both tibias. Similar observations were made by Johari et al. who observed one child with bilateral tibial involvement [1].

The left side was more involved than the right side, and more girls than boys were involved.

### Resolution of bowing and shortening

Dawson et al. performed tibial osteotomy to correct deformities as early as 12 months of age [13]. At 5 years, they noticed that the posterior bow had remodelled fully, but the medial bow had persisted. Miller et al. presented similar findings in their study [14]. Shah et al. reported that the rate of remodelling of the posterior bow was greater than that of the medial bow [3]. They observed faster physeal remodelling than diaphyseal remodelling. Wright et al. and Gordon et al. reported similar findings in which the posterior bow remodelled faster than did the medial bow [4]. Sevencan et al. reported that bows resolve at a parallel rate [6] (Table 13).

In this study, the remodelling of the posterior diaphyseal bow was significantly greater than that of the medial or oblique diaphyseal bow. The rate of overall remodelling was 0.6 degrees/month. The posterior diaphyseal bow was corrected by 65% of its value at presentation. The inter-physeal bow in the lateral plane was reduced by 70% of its value at presentation. The rate of remodelling of the inter-physeal bow in the lateral plane was half that of the remodelling rate of the posterior diaphyseal bow. This faster remodelling could be explained by the Wolffes law and Heuter–Volkans law, which suggest faster correction along the axis of movement of the joint and along the line of stress over the tibia.

The medial diaphyseal bow and inter-physeal bow in the antero-posterior plane resolved to 60% of their value at presentation. The rates of remodelling of these parameters were significantly different.

**Table 13:** Summary of the literature

References	N	Pattern of resolution of deformity	Correlation between remodeling of deformity and length	Method used for estimating tibial shortening at maturity	Age at which tibial shortening at maturity was predicted and estimated amount of shortening
Yadav et al. [15]	6	–	–	−	–
Hofmann et al. [16]	13	Angular deformities resolve slowly	Direct relationship between degree of angulation and severity of leg length discrepancy	–	–
Pappa et al. [7]	33	Resolution was rapid in first 3 years of life Posterior bow remodeled faster than the medial bow	Degrees of angulation correlated positively with tibial shortening Proportionate shortening remained stable and followed type 1 Shapiro curve	Measured from proportionate tibial length	Shortening estimated at 3 years was accurate up to 2 mm at maturity
Shah et al. [3]	20	Rapid remodeling in first year of life Remodeling of posterior bow was greater than medial bow	More severe the medial bowing, the greater the degree of shortening Posterior bow remodeled faster than the medial bow	–	–
Johari et al. [1]	20	Medial and posterior angulations reduced as child grew	–	–	–
Kaufman et al. [17]	11	–	–	–	–
Napiontek et al. [18]	4	–	–	–	–
Ariyawatkul et al. [19]	4	–	–	Not mentioned	Expected LLD at maturity: 5.1–9.9 cm
Zivanovic et al. [20]	6	Full correction of medial bow, partial correction of posterior bow	–	–	–
Wright et al. [4]	38	Posterior bow greater than the medial bow	Correlation between severity of posterior bow and LLD%	–	Recommended LLD at maturity to be estimated at 4 years to develop a surgical plan as the quality of radiographs and absolute size of the patient would be better for estimation
Di Gennaro et al. [5]	44	Posterior bow corrected more than medial bow	Greater the angular deformity at birth, wider the LLD at maturity	–	4.3 cm at maturity (LLD% 13%) Age not mentioned
Gordon et al. [10]	16	–	–	Green Anderson method Moseley method Mutiplier method	9 years and 5 months (range: 3 years and 2 months to 14 years)
Sagade et al. [9]	23	–	–	Moseley method (before 2000) Multiplier method (After 2000)	Divided into two groups < 5 years–7.2 cm estimated shortening at maturity > 5 years–5 cm estimated shortening at maturity
Sevencan et al. [6]	22	Resolution of medial bowing was parallel to posterior bowing	Greater the degree of bowing in first year of life, greater the LLD Resolution of LLD followed type 1 Shapiro curve (congenital type)	Green Anderson and Multiplier method	–
Johari et al. [21]	22	–	–	Not mentioned	Divided into two groups < 10 years: 4.9 cm estimated shortening at maturity > 10 years: 5.17 cm estimated shortening at maturity
Current study	51	Posterior diaphyseal bow remodeled faster than medial and oblique diaphyseal bows	Remodeling of oblique plane deformity showed maximum corelation with resolution of LLD	Multiplier method	Tibial shortening at maturity estimated at first presentation and every subsequent follow-up Estimated shortening at maturity at final follow-up: 6.7 ± 2.3 cm

N = Number of study participants, LLD = Limb length discrepancy

The oblique bows of the diaphysis and the inter-physeal plane resolved to 60% of their original values. The rates of remodelling in these bows were similar (0.45/month).

There was a significant difference between the rates of remodelling at the diaphyseal and physeal levels in both the antero-posterior and lateral planes. The remodelling of the diaphyseal bows was greater than that of the physeal bows.

Heyman et al. and Miller et al. reported varied amounts of tibial shortening in their series and reported that shortening is a nonsevere issue in CPMBT that corrects itself in time [14, 22]. The first detailed analysis of tibial shortening was performed by Hofman et al. who reported that shortening worsens over time at a rate of 1.5 mm to 1 cm [16]. Pappa et al. reported that the percentage of tibial shortening remains constant and stable throughout and does not change [7]. Wright et al. reported similar lines with a constant rate of 14% tibial shortening throughout [4]. Giovanni et al. reported that the final shortening was 4.3 cm greater than the initial shortening, but the percentage shortening remained constant at 13% [5]. De Maio et al. postulated that the unbalanced stresses on the physis caused growth inhibition, and hence, shortening occurred from an early age.

In this study, the amount of shortening increased from 2 cm at presentation to 4 cm at the final follow-up [8]. This corresponded to a 40% increase in the magnitude of tibial shortening. The percentage shortening of the affected tibia compared with the normal tibia decreased from 20 to 13% at 6 years of age, after which it remained similar (Fig. 6). This did not follow the type 1 Shapiro curve, as mentioned by previous authors [4, 7, 9, 10]. The rate of overall remodelling was 0.07%/month. This corresponded to 35% reduction in the percentage of shortening at the final follow-up.

### Yearly remodelling of deformities and shortening

Wright et al. studied the yearly rate of remodelling of the medial diaphyseal and posterior diaphyseal bows [4]. They observed the highest rate of remodelling in the first year of life and mentioned that the remodelling ceased after 4 years of age. These findings were similar to those of Shah et al. [3].

In this study, the greatest amount of remodelling occurred in the first 12 months of age. During this period, the posterior bow remodelled the fastest. This rate of remodelling decreased to almost half of its value between 1 and 2 years and halved further between 2 and 3 years. The rates of remodelling at these times were significantly different. After 3 years, the remodelling rates were similar, and remodelling continued up to 6 years of age. After 6 years of age, minimal remodelling was observed. The percentage shortening also remodelled fastest in the first year of life. This halved in the second year and further halved thereafter up to 6 years of age. After 6 years, the degree of shortening was minimal.

At 6 years of age, the medial diaphyseal bow remodelled to 10.5% and the, to 9% of its index value at presentation at 1 year of age. The posterior diaphyseal bow remodelled to 5.5%, and the lateral inter-physeal bow remodelled to 10% of its value. These values provide predictive models of the residual bow at 6 years of age and aid surgeons in planning deformity correction procedures as early as 1 year of age.

In the CPMBT, there is significant controversy around the timing of deformity correction and tibial length equalization. Some authors [1, 4, 21] have advocated against early intervention and reserved it for a small group of children who have severe deformities. However, other authors [9] have mentioned that early intervention would add to the length correction. However, the correct age at which one should intervene has not been mentioned.

In this study, we investigated the natural history of the remodelling of tibial deformities and tibial shortening. We suggest addressing the deformity and shortening at an age not less than 6 years. This would allow natural remodelling of the length and tibial shortening processes, after which the surgeon could call for any intervention if needed. Severe deformities or tibial shortening, as mentioned by Johari et al. could be addressed at an age greater than 6 years, which would allow simultaneous correction of deformity and tibial length [21]. However, this would require caution, as lengthening may relapse, and repeat procedures may be needed later, close to maturity.

### Correlation between the rate of deformity remodelling and the rate of limb shortening correction

Shah et al. reported that there was a significant correlation between the severity of the posterior bow and the severity of shortening [3]. Wright et al. reported similar findings, but they reported that the contributions of oblique plane deformities could be significant, but they studied this further [4]. Giovanni et al. reported a significant correlation between the inter-physeal angle in the antero-posterior plane and tibial shortening [5].

In this study, the maximum correlation was found between the rate of remodelling of tibial shortening and the rate of remodelling of the oblique plane deformities in the diaphysis and the inter-physeal level. This finding establishes that CPMBT is a true triplanar deformity with a significant oblique bow that affects the correction of tibial shortening. Thus, while planning surgical treatment for this condition, the oblique bow must be assessed and marked, and the deformity must be corrected in this plane to achieve tibial length equalization.

### Prediction of tibial shortening with the multiplier method

Pappa et al. estimated tibial shortening at maturity on the basis of proportionate tibial lengths. The authors suggested that tibial shortening follows a type 1 Shapiro curve and that shortening at maturity can be accurately measured at 3 years of age [7]. Wright et al. reported that tibial shortening in the CPMBT followed a Shapiro type 1 curve and suggested that the final shortening could be predicted on population-based data [4]. The authors suggested that tibial shortening at maturity be assessed at four years of age, as the size of the patient and the quality of the radiographs would be better. Gordon et al. used the Green‒Anderson method, the Moseley straight line graph and the multiplier method to assess final tibial shortening [10]. They estimated it to be approximately 4 cm at maturity. However, the exact age at which it could be accurately predicted has not been specified. Sagade et al. also used the multiplier method to predict tibial shortening at maturity. They divided their study cohort into early (< 5 years) and late lengthening groups (> 5 years) but did not specify the exact age at which shortening at maturity could be predicted [9]. Johari et al. also divided their study cohort into two groups: early intervention (< 10 years) and late intervention (> 10 years) [21]. They did not find any significant difference between the estimated shortening in either group. However, the age at which it was measured has not been specified.

In this study, the multiplier method [11] was used to assess the degree of predicted tibial shortening at follow-up. Tibial shortening did not follow the type 1 Shapiro curve pattern. There was a significant difference between the tibial shortening predicted at the initial presentation and the predicted tibial shortening at the final follow-up. When analyzed further, there was a significant difference between the predicted tibial shortening up to 2 years of age, after which the predicted tibial shortening was similar.

This analysis clarifies the age at which the multiplier method can be used. After 2 years of age, final tibial shortening may be predicted, and depending on the magnitude of estimated tibial shortening at maturity, procedures for tibial length equalization, such as opposite tibial epiphysiodesis or tibial lengthening, can be planned, and parents can be counseled accordingly.

## Conclusions

1. Remodelling of the posterior diaphyseal bow was greater than that of the medial or oblique diaphyseal bows.
2. The magnitude of tibial shortening increased with the growth of the child; however, the percentage of shortening of the tibia remodelled from 20 to 13% at the final follow-up.
3. The highest degree of deformity remodelling occurred in the first year of life and decreased significantly up to 3 years of age. After 3 years, the remodelling occurred at similar rates up to 6 years, after which there was negligible resolution.
4. In children with severe deformities, deformity correction or tibial length correction procedures could be planned after 6 years of age as the resolution reaches a plateau.
5. The highest correlation for percentage remodelling of tibial shortening was observed with deformity resolution in the oblique plane.
6. Tibial shortening at maturity could be predicted using the multiplier method after 2 years of age.

## Limitations

- The outcomes of the surgical interventions performed were not evaluated.
- Only a small proportion of the children reached skeletal maturity.

## Strengths

- This is the largest cohort of patients with CPMBT with good follow-up with detailed insight into the natural history of remodelling of deformities.
- The oblique plane of deformities has been described in detail. This provides valuable insights during planning surgery.

## Implications

- Parents of children suffering from congenital posteromedial bowing of the tibia can be counselled for the exact natural history of deformity and the resolution of tibial shortening.
- Limb shortening at skeletal maturity can be accurately predicted after 2 years of age.

## Data Availability

The datasets used and/or analyzed during the current study are available from the corresponding author upon reasonable request.

## Abbreviations

CPMBT: Congenital posteromedial bowing of the tibia
AP: Antero-posterior

## References

[1] Johari AN, Dhawale AA, Salaskar A, Aroojis AJ. Congenital postero-medial bowing of the tibia and fibula: is early surgery worthwhile? J Pediatr Orthop B. 2010;19(6):479–86. 20613643 10.1097/BPB.0b013e32833ccac2

[2] Rathgeb JM, Ramsey PL, Cowell HR. Congenital kyphoscoliosis of the tibia. Clin Orthop Relat Res. 1974;103:178. 10.1097/00003086-197409000-00079 4606380

[3] Shah HH, Doddabasappa SN, Joseph B. Congenital posteromedial bowing of the tibia: a retrospective analysis of growth abnormalities in the leg. J Pediatr Orthop B. 2009;18(3):120–8. 19339901 10.1097/BPB.0b013e328329dc86

[4] Wright J, Hill RA, Eastwood DM, Hashemi-Nejad A, Calder P, Tennant S. Posteromedial bowing of the tibia: a benign condition or a case for limb reconstruction? J Child Orthop. 2018;12(2):187–96. 29707059 10.1302/1863-2548.12.170211 PMC5902754

[5] Di Gennaro GL, Gallone G, Martinez Vazquez EA, Marchesini Reggiani L, Racano C, Olivotto E, et al. Deformity progression in congenital posteromedial bowing of the tibia: a report of 44 cases. BMC Musculoskelet Disord. 2020;3(21):430. 10.1186/s12891-020-03408-w PMC7334844 32620101

[6] Sevencan A, Ucpunar H, Akgun H, Ozyalvac ON, Akpinar E, Bayhan AI, et al. Evolution of the angular deformity and limb length discrepancy of congenital posteromedial bowing of the tibia over time. Jt Dis Relat Surg. 2022;33(3):567–73. 36345184 10.52312/jdrs.2022.718 PMC9647689

[7] Pappas AM. Congenital posteromedial bowing of the tibia and fibula. J Pediatr Orthop. 1984;4(5):525. 6490868

[8] De Maio F, Corsi A, Roggini M, Riminucci M, Bianco P, Ippolito E. Congenital unilateral posteromedial bowing of the tibia and fibula: insights regarding pathogenesis from prenatal pathology: a case report. JBJS. 2005;87(7):1601. 10.2106/JBJS.D.02551 15995131

[9] Sagade B, Jagani N, Chaudhary I, Chaudhary M. Congenital posteromedial bowing of tibia: comparison of early and late lengthening. J Pediatr Orthop. 2021;41(9):e816–22. 34387229 10.1097/BPO.0000000000001935

[10] Gordon JE, Schoenecker PL, Lewis TR, Miller ML. Limb lengthening in the treatment of posteromedial bowing of the tibia. J Child Orthop. 2020;14(5):480–7. 33204357 10.1302/1863-2548.14.200111 PMC7666791

[11] Paley D, Bhave A, Herzenberg JE, Bowen JR. Multiplier method for predicting limb-length discrepancy. J Bone Joint Surg Am. 2000;82(10):1432–46. 11057472 10.2106/00004623-200010000-00010

[12] Paley D. Principles of deformity correction. Springer science & Business Media. 2002;860

[13] Dawson GHJ. Intra-uterine fractures of the tibia and fibula: report of a case with correction by osteotomy and plating. J Bone Joint Surg Am. 1949;31(2):406. 18125309

[14] Miller BF. Congenital posterior bowing of the tibia with talipes calcaneo-valgus. J Bone Jt Surg Br. 1951;33(1):50–5. 10.1302/0301-620X.33B1.50 14814160

[15] Yadav SS, Thomas S. Congenital posteromedial bowing of the tibia. Acta Orthop Scand. 1980;51(2):311–3. 7435191 10.3109/17453678008990804

[16] Hofmann A, Wenger DR. Posteromedial bowing of the tibia. Progression of discrepancy in leg lengths. J Bone Joint Surg Am. 1981;63(3):384–8. 7204436

[17] Kaufman SD, Fagg JA, Jones S, Bell MJ, Saleh M, Fernandes JA. Limb lengthening incongenital posteromedial bow of the tibia. Strat Trauma Limb Reconstr. 2012;7(3):147 10.1007/s11751-012-0145-4 PMC3482434 23070867

[18] Napiontek M, Shadi M. Congenital posteromedial bowing of the tibia and fibula: treatment option by multilevel osteotomy. J Pediatr Orthop B. 2014;23(2):130–4 24390537 10.1097/BPB.0000000000000024

[19] Ariyawatkul T, Kaewpornsawan K, Chotigavanichaya C, Eamsobhana P. The Results of Lengthening in Congenital Posteromedial Angulation of Tibia. J Med Assoc Thai. 2016;99(10):1137–41 29952465

[20] Živanović D, Slavković A, Marjanović Z, Djordjević I, Bojović N, Petrović M. Congenital Posteromedial Bowing of the Tibia: A Single Center Experience. Acta Fac med Naiss. 2017;34(4).

[21] Johari AN, Anjum R. Lengthening in congenital posteromedial bowing of tibia: a follow-up series at skeletal maturity. Int Orthop. 2024;48(6):1439–52. 38594586 10.1007/s00264-024-06160-1

[22] Heyman CH, Herndon CH, Heiple KG. Congenital posterior angulation of the tibia with talipes calcaneus: a long-term report of eleven patients. J Bone Joint Surg Am. 1959;41(3):476. 13641299

